# Comparative molecular docking analysis of the SARS CoV-2 Spike glycoprotein with the human ACE-2 receptors and thrombin

**DOI:** 10.6026/97320630016532

**Published:** 2020-07-31

**Authors:** Piyush Bhanu, Nisha Hemandhar Kumar, Shamini Hemandhar Kumar, Maitrali Relekar, Daniel Alex Anand, Jitendra Kumar

**Affiliations:** 1 Xome Life Sciences, Bangalore Bioinnovation Centre, Helix Biotech Park, Bengaluru, Karnataka-560100,India; 2 Director, Bangalore Bioinnovation Centre (BBC), Helix Biotech Park, Electronics City Phase 1, Bengaluru, Karnataka-560100,India; 3 Department of Bioinformatics and The Centre for Molecular Data Science and Systems Biology, Sathyabama Institute of Science and Technology, Chennai, Tamil Nadu-600119,India; 4 School of Computing, Newcastle University, Newcastle upon Tyne NE1 7RU, United Kingdom

**Keywords:** SARS CoV-2 spike glycoprotein, Thrombin, ACE-2, molecular interaction

## Abstract

Comparative molecular docking and vixualization analysis of the human thrombin with the SARS CoV-2 Spike glycoprotein and the human ACE-2 receptors is of interest. The data shows that residues spanning positions 30-41 in the ACE-2 have interaction with the spike glycoprotein (UniProt ID: Q9BYF1). Results also shows that thrombin binds with SER494 in the spike protein, and GLU37 in the ACE2 receptor. SER494 in the viral receptor-binding domain provides support for hotspot-353 reported elsewhere. These preliminary data provide insights for further probe.

## Background

A viral infection of second Severe Acute Respiratory Syndrome Coronavirus (SARS-CoV-2), originated in China in December 2019, has spread across the world rapidly. [1] More than seventeen lakh people have been diagnosed from 213 countries, out of which 106,138 have died. [2] Hence, there is an urgent need to find a way to put an end to this pandemic outbreak. Coronaviruses have infected humans causing lethal pneumonia by crossing species barriers: severe acute respiratory syndrome coronavirus (SARS-CoV) in 2003 [3], and Middle-East respiratory syndrome coronavirus (MERS-CoV) in 2012. [4] SARS-CoV uses angiotensin-converting enzyme-2 (ACE-2) cell receptors to infect host cells, whereas MERS-CoV enters via CD26. [5] Receptor-binding domain (RBD) of SARS-CoV is structurally similar to the RBD of SARS-CoV-2 based on molecular modelling studies. [5] SARS-CoV-2 spike glycoprotein has 76% identity with the SARS-CoV and 80% with bat SARS-CoV ZXC21 S and ZC45 S-glycoprotein. [6] Therefore, it was suggested that carboxypeptidase ACE-2, an integral membrane glycoprotein of the human host cell, is used as an entry point by the virus [7].

The ACE-2, a type 1 integral membrane glycoprotein, is expressed highly in the kidney, the heart and the lungs and is also elevated in the neurons and the glial cells of the brain, blood vessels and intestine. [8] ACE2 is of crucial importance in the physiological modulation of the renin-angiotensin system, a cell-signalling pathway. [9] ACE-2 metabolises angiotensin II and produces peptides angiotensin (1-7), which help in the widening of the blood vessels. [10] It plays a critical role in various diseases like diabetes, renal impairment and cardiovascular diseases. [10] Thrombin is a coagulation protein in the bloodstream, which converts soluble fibrinogen into insoluble strands of fibrin as well as catalysing many other coagulation-related reactions. [11] It also causes secondary effects to thrombosis; such as proliferation of vascular smooth muscle cells and fibroblasts, chemotaxis of monocytes and adhesion of neutrophils. [11] In 2011, Simmons et al. found out that infectivity of viruses having wild type SARS-spike protein was reduced to a certain extent because of thrombin.[12] In this computational study, our objective is to explore the interaction of thrombin with the spike-glycoprotein using molecular docking. Hence, we describe the predicted interactions between SARS-CoV-2 spike glycoproteins and the human thrombin to infer the mechanism of viral entry using molecular docking analysis followed by molecular visualization of gleaned binding data.

## Methods:

Docking of SARS-CoV-2 spike glycoprotein (PDB ID: 6VXX) [6] and ACE-2 human cell receptor (PDB ID: 6VW1) was completed using data downloaded from RCSB-PDB. [13] We used MetaPocket [14] to identify the potential ligand binding sites in the protein on initializing the number of pockets to three. AUTODOCK4 was used to predict the molecular interaction between the small molecule (thrombin) and macromolecule targets (6VXX and 6VW1) [15].

Initially, a grid box was set up around the binding site or the active site of the protein. It is an essential step to perform molecular docking of ligand and receptor. On clicking the grid option on the ADT interface, a dialog box for grid option appeared. Furthermore, the coordinate values and default parameters were entered. Number of points was modified for X, Y, and Z dimensions by rotating the dial. The grid box should be large enough to accommodate the ligand in its extended conformation. Finally, the optimization of the protein and the ligand was saved in .pdbqt format. The further steps for docking were carried out using the protocol [16] and the resultant complex.pdb file was visualized using PYMOL [17] after which, pose with lower binding energy was selected.

## Results and discussion:

### Binding of Thrombin with the spike-glycoprotein (6VXX):

The PDB deposited electron microscopy (EM) structure 6VXX has a resolution of 2.80 Å, with no Ramachandran outliers. The EM structure has fifty-nine potential ligand binding sites as predicted by MetaPocket. On performing the docking for SARS-CoV-2 spike glycoprotein (6VXX) and thrombin using AUTODOCK4, the top three poses with lower binding affinity were selected. The 2nd pose has a binding affinity of -5.22 kcal/mol and 153.36 RMSD, with atom H17 of the ligand with O of SER390 in the spike protein with a bond length of 2.6Å. There is another interaction with atom H25 of the ligand with O of PRO403 in the protein with a bond length of 2.2Å, and an interaction with atom H18 of the ligand and O of the protein with a bond length of 1.9Å. Furthermore, an interaction for the 4th pose has a binding affinity of -5.11 kcal/mol and 144.17 RMSD, with atom H24 of the ligand with O of ASN456 in the spike protein with a bond length of 1.8Å. There is another interaction with atom H25 of the ligand with OD1 of ASN459 in the protein with a bond length of 2.1Å, and an interaction with atom H18 of the ligand and OG1 of THR364 in the protein with a bond length of 3.5Å. An interaction for the 7th pose has a binding affinity of -4.93 kcal/mol and 144.40 RMSD, with atom H24 of the ligand with O of ASN456 in the spike protein with a bond length of 1.7Å. There is another interaction with atom H25 of the ligand with OD1 of ASN459 in the protein with a bond length of 2.1 Å, and an interaction with atom H18 of the ligand and OG1 of THR364 in the protein with a bond length of 2.1Å.

### Binding of Thrombin with the ACE-2 receptor:

**Figure 1:**
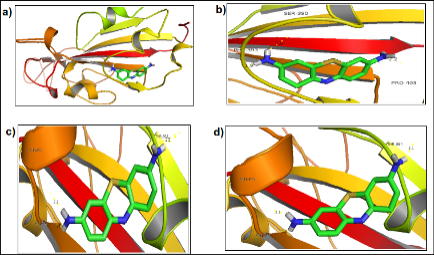
Intermolecular interactions of thrombin with amino acids in the spike protein (6VXX) (visualized using pymol). Figure 1a: Structural representation of 6VXX and thrombin, Figure 1b: Molecular interaction of thrombin with the 2nd pose, Figure 1c: Molecular interaction of thrombin with the 4th pose, Figure 1d: Molecular interaction of thrombin with the 7th pose, Key - the sticks represents thrombin, the secondary structure represents the spike protein, dotted lines represents the interactions between the protein and ligand.

**Figure 2:**
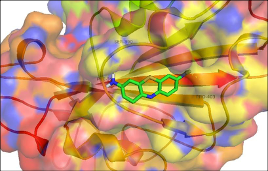
Intermolecular interaction of thrombin with amino acids at active sites in the spike protein (6VXX) (visualized using pymol).

**Figure 3:**
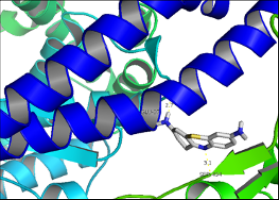
Intermolecular interaction of thrombin with amino acids in 6VW1 protein (visualized using pymol). Key - the sticks represent the thrombin, the secondary structure represents the Structure of SARS CoV-2 chimeric receptor-binding domain (green) complexed with its receptor human ACE2 (blue), dotted lines represent the interactions between the protein and ligand.

The PDB deposited crystal structure 6VW1 has a resolution of 2.68Å, R-free value of 0.228 and with no Ramachandran outliers. On performing the docking for SARS CoV-2 chimeric receptor-binding domain complexed with its receptor human ACE2 (6VW1) and thrombin using AUTODOCK4, the pose with an interaction with ACE and RBD domain was selected. The protein-ligand docking performed for the ligand thrombin and protein 6VW1, shows an interaction with binding affinity -4.55 kcal/mol and 118.99 RMSD, with atom H16 of the ligand with O of GLU35 in the ACE2 receptor of 6VW1 with a bond length of 2.7Å. Furthermore, there is another interaction with atom N6 of the ligand with OG of SER494 in the RBD domain of the protein with a bond length of 3.1Å. In accordance with the docking results for 6VXX and thrombin (Figure 1,Table 1) all three poses, which were selected based on the binding affinity, have an interaction with the potential ligand binding sites as predicted by MetaPocket. However, pose 2 has a lower binding energy and thrombin has an interaction with PRO403 in the binding pocket of the spike glycoprotein (Figure 2). Consequently, pose 2 could have a more stable protein-ligand complex and, an impact in inhibiting the binding of spike glycoprotein to the ACE-2 receptor of the cellular membrane. The residues L455, F486, Q493, S494, N501 and Y505 of the receptor-binding domain in the spike protein are the most likely binding sites for the ACE2 receptor. [18] Furthermore, the amino acid positions spanning from 30 - 41 are having an interaction with spike glycoprotein (UniProt ID: Q9BYF1). [19] The docking results for 6VW1 and thrombin, illustrate that thrombin binds with SER494 in the spike protein and GLU37 in the ACE2 receptor. (Table 2, Figure 3). It has been shown conclusively that SER494 in SARS CoV-2 receptor binding domain provides support for hotspot - 353. In the overall big picture, the results we have obtained in this study include LEU455, PHE486 and SER494 of the receptor binding domain support that SARS CoV-2 uses human ACE-2 and can infect human cells. These interactions may mediate conformational changes in the viral spike proteins that are critical for entry or activation of signalling pathways that are key to the infectious life cycle of the pathogen.

## Conclusions:

We describe the comparative molecular docking analysis of the SARS CoV-2 Spike glycoprotein with the human ACE-2 receptors and thrombin to probe further in the combat against covid-19 viral outbreak.

## Figures and Tables

**Table 1:** Molecular interactions of thrombin with amino acids in the spike protein (6VXX)

PDB ID	Ligand	Binding Pose	Binding Energy (Kcal/mol)	RMSD	Receptor	Bond Length (Å)
6VXX	Thrombin	2	-5.22	153.36	SER390	2.6
					PRO403	2.2
					PHE393	1.9
		4	-5.11	144.17	ASN456	1.8
					ASN459	2.1
					THR364	3.5
		7	-4.93	144.4	ASN456	1.7
					ASN459	2.1
					THR364	2.1

**Table 2:** Molecular interactions of thrombin with amino acids in protein with PDB ID: 6VW1

PDB ID	Ligand	Binding Pose	Binding Energy (Kcal/mol)	RMSD	Receptor	Bond Length (Å)
6VW1	Thrombin	1	-4.55	118.99	GLU37	2.7
					SER494	3.1
